# Digital image management in a globalised world: opportunities and challenges

**DOI:** 10.2349/biij.4.4.e26

**Published:** 2008-10-01

**Authors:** BJJ Abdullah

**Affiliations:** Department of Biomedical Imaging, Faculty of Medicine, University of Malaya, Kuala Lumpur, Malaysia

Good health for all populations is an accepted international goal [1], and there have been broad gains in life expectancy over the past century. However, the perspectives of how health is best defined vary considerably, and health inequalities between the rich and the poor persist. The prospects for future health depend increasingly on the relatively complicated process of globalisation [2]. In 2001, the WHO Commission on Macroeconomics and Health demonstrated that health is not only a benefit of development, but is also indispensable to development [3]. Illness all too often leads to "medical poverty traps" [4], creating a vicious circle of poor nutrition, forgone education, and still more illness. All of these then undermine the economic growth that is necessary, although not sufficient, for widespread improvements in health status.

The promotion of health equity is defined in the literature as "the absence of disparities in health (and in its key social determinants) that are systematically associated with social advantage/disadvantage". The Social Determinants of Health (CSDH), established by the WHO, are considered the fairest and most effective way to improve health for all people and reduce inequalities. Social determinants of health, broadly stated, are the conditions under which people live and work which affect their opportunities to lead healthy lives. Good medical care is vital, but unless the root social causes that undermine people's health are addressed, the opportunity for well-being will not be achieved.

Globalisation is a term with multiple hotly contested definitions and meanings. And it is generally an umbrella term for a complex series of economic, social, technological, cultural and political changes across the globe. The most appropriate definition of globalisation would be "a process of greater integration within the world economy through movements of goods and services, capital, technology and (to a lesser extent) labour, which leads increasingly to economic decisions being influenced by global conditions" [5]. The outcome of globalisation is the increasing interdependence and interaction among people, companies, and governments of different nations, driven by international trade and made possible by innovations in information technology.

Historically, companies in developed countries have led globalisation by pushing products and services into developing countries and emerging economies. Now, emerging countries are pushing their services into developed countries with quality labour and services at low cost. In short, globalisation has become a two-way street as both sides exploit each other's markets and economies, creating a virtual world labour and market force. [6]. Even though globalisation is most often seen as an economic process, the current view of globalisation is that of a more comprehensive phenomenon. This phenomena is being shaped by a multitude of factors and events but at the same time is reshaping our society rapidly [7-9].

Thus the emergence of the global marketplace has taken on the following features [10]:

- New global governance structure – Globalisation influences the interdependence among nations as well as the nation state's sovereignty leading to (a need for) new global governance structures.
- Global markets – Globalisation is characterised by worldwide changes in economic infrastructures and the emergence of global markets and a global trading system.
- Global communication and diffusion of information – Globalisation makes the sharing of information and the exchange of experiences around common problems possible.
- Global mobility – Global mobility is characterised by a major increase in the extensity, intensity and velocity of movement and by a wide variety in 'types' of mobility.
- Cross-cultural interaction – Globalising cultural flows result in interactions between global and local cultural elements.
- Global environmental changes – Global environmental threats to ecosystems include global climate change, loss of biodiversity, global ozone depletion and the global decline in natural areas.

While some may argue that globalisation allows poor countries and their citizens the opportunity to raise their standards of living, encourage democracy and embrace multiculturalism, others claim that globalisation has simply allowed Western corporations to overwhelm world markets at the expense of small businesses, local cultures, traditions and values. Globalisation should not be seen as a process that is inherently “bad” or “good” but rather, a process capable of both positive and negative outcomes. The outcomes of globalisation are entirely dependent on how policies are guided and implemented. The promotion and resistance of globalisation has taken shape at both a popular and governmental level. It is believed that such efforts can only hope to steer globalisation, not alter it.

Contemporary globalisation has been promoted, facilitated and not uncommonly enforced by political choices about such matters as trade liberalisation, financial (de)regulation, provision of support for domestically headquartered corporations [11] and the conditions under which development assistance is provided. Sadly, globalisation, to a large extent, appears to have economically benefited the heavily industrialised countries with serious adverse consequences for developing nations, with some notable exceptions. Several national economies collapsed under the forced introduction of global market economics through conditional loans granted by the International Monetary Fund [the IMF is incidentally the primary lender of money to developing countries]. This was evident during the Asian Financial Crisis of 1997. However, China chose to implement its own non-IMF dictated path towards a market economy which highlighted that implementation and guidance of a winning policy makes or breaks the results of globalisation. What is even more important to recognise is that they are alternative pathways for developing countries, using non-IMF dictated policies, to allow a more gradual approach to introducing market economics. Such notable successes would go a long way in allowing developing nations to claim a stake on the potential profits of globalisation.

In the medical domain, irrespective of the state of development, globalisation has resulted in the increased speed with which information about new treatments, technologies and strategies for health promotion can be diffused. There are also more opportunities for enhanced political participation and social inclusion that are offered by new, *potentially* widely accessible forms of electronic communication. However, it must be recognised that it has been the “economic aspects of globalisation” of healthcare that has been the driving force behind the overall process of globalisation over the last two decades" [10]

Indeed the accrued benefits of globalisation vary between the developed and less-developed nations and, as a result, globalisation of healthcare raises some more serious issues, which are:

- How can countries deal with globalisation in the context of their existing cultures, beliefs, resources and systems?
- How do we deal with the impact of globalisation on the healthcare delivery systems of the various jurisdictions [12]?
- How does one cope with the massive impact on a country’s economy of claiming a share of the economic activity that the health industries and service sectors represent, which incidentally is the largest industry on a global scale [12]?
- How can developing nations protect their indigenous treatments from being patented in industrialised nations?
- How can the serious brain drain of the limited healthcare personnel from developing nations toward the industrialised West be moderated to ensure the needy have access to the services of this invaluable resource?
- What mechanisms should be in place to ensure universal access to essential medication and basic imaging facilities?

Medical imaging may not be the only productivity driver as digital imaging and information technology (IT) allow providers to better manage vast volumes of data at a lower cost. When one looks at the benefits of health IT, the logic behind such IT-intensive investments include efficiency gains, such as enhanced transaction speed, reduced labour costs associated with the administrative tasks – which can make up 25 percent of the overall cost – improved patient safety, privacy and access to information. The increased competitive environment, with the commodification of healthcare has also resulted in the thinning profit margins putting the very survival of healthcare facilities to the test. Many are betting that IT can produce the efficiency gains and compensate for dwindling reimbursements. ROI [return-on-investment] of 10 percent with increasing overall efficiency of 13 to 15 percent have been shown when the PACs have been employed.

The prevailing perception among hospital administrators and physicians is that implementation of IT will also provide a competitive advantage. The better use of technology and interoperable electronic networks should accelerate integration, standardisation and knowledge transfer of the administrative and clinical information, especially in the context of the globalisation of healthcare and efforts in many countries to create a sustainable health system [13].

With the increased competitiveness in the healthcare industry, both nationally and regionally, the issue of data management is a vital consideration for success in this globalised digital world. Only two elements exist in a connected world: the customer and the information [14]. The key to the former lies in managing the latter. The use of data mining of electronic patient data generated by IT systems such as PACS and RIS, along with analysis/trending by charge codes, has also been promoted to help justify healthcare’s increasing reliance on medical imaging. There is a trend toward using IT solutions and sophisticated practice management tools, be they Web solution, real-time data mining or dashboards, to help them manage their practice as a business. This would make key business data seamlessly available for decision support that could cut costs by identifying and managing process inefficiencies and track revenues to improve business. This would allow organisations to track revenues by exams vs. charges; planned vs. actual scheduling; referring physician trends – where are referrals coming from?; referral market trends – measure referrals by zip codes and demographics; staff efficiency – average and individual, track productivity – volume trends; incomplete exam work – unfinished exams affect your bottom line and efficiency; accounts receivables – average payment periods; and total patient time – how long does it take to get the report to the referring physician? [15]. Ultimately, it is hoped that such use of data-mining would result in better outcomes, more cost-effective processes and overall improved healthcare. The current scenario where the information is stored in different “silo” or databases with different software and operating systems makes this all the more impossible.

It has been promoted that information technology will allow a significant proportion of healthcare services to be offshored. Many of the leading healthcare technology vendors have significant or growing overseas operations, while others are contracting for these services. The future of healthcare outsourcing and offshore services will vary across the provider, payer, and supplier sectors. The jury is still out, since major challenges occur when one looks at issues of cross-border transfer of digital image information, whether for purposes of reading or management. This is, in part, due to the highly regulatory environment and national compliance requirements. The promotion of cost reduction and savings for using offshore services in healthcare should not by themselves be solid business grounds for offshoring. It is essential that organisations consider the use of offshore services as a strategic tool which must be integrated with their business model e.g. would these offshore services support and improve the organisation's overall ability to deliver quality services? Would patient safety or improved care delivery efficiencies and service levels get better?

On the downside, the implementation of such sophisticated healthcare information technology systems are a heavy initial investment that come at a hefty price. Several solutions have been proposed to overcome this. The use of a Web-based platform or application service provider solutions may be a way for organisations to cost-effectively utilise these solutions to provide high-quality patient care. Application service provider solutions shift the burden of technical support away from the organisation, allowing them to focus on their core competency of healthcare so as to spread out the costs over an extended period of time. Upgrades or changes to the systems to stay current come with risks and inconvenience to the users who have to learn to use a variety of different log-ins, platforms and formats to access the data. In addition, the increased frenzy of mergers and acquisitions between organisations, big and small, raise additional issues of integration of the digital management systems. This further adds to the cost and complexity of managing the outcomes with a possible decrease in the quality of services of the smaller entities as those systems are slowly phased out with no possibility of any upgrades.

Even though branding is very often used to sell products with no real value beyond that perceived by the buyer, the promotion of vital and good technology i.e. digital image management, in the bigger picture may be pointless unless its “brand” is perceived to be of value and requires the necessary buy-in from governments, professionals, managers and the public. Therefore the use of price as the sole criterion for success in the information-based industry may not be enough [14] as the tools for conducting business electronically are low. Even though the image reads being performed by expert radiologists in India of images from either the US or UK may be as good, if not better, than those of their countries of origin, the lack of “branding” by the offshore readers is a major hindrance resulting in numerous unsubstantiated claims. This is in contrast to the image reads being done in Australia, for instance, where these same issues are not raised.

With the growing trend in healthcare toward higher operational costs, reduced reimbursement and heightened competition among imaging service providers, healthcare facilities must carefully plan capital equipment acquisitions and budgets. Many of today’s high-end modalities such as CT, MRI and PET/CT are multi-million dollar purchases that require strategic planning for implementation as well as detailed marketing initiatives that can help maximise utilisation. Increasing the efficiency of radiology – from the technical to the professional component – is a key factor in maximising utilisation of nearly all diagnostic imaging modalities. Accomplishing more with less, particularly with a shortage of radiologists, and in some areas, technologists is an operational reality. Data mining RIS/PACS and CPT code analysis may help justify healthcare’s increasing reliance on medical imaging, despite reduced reimbursements [16].

A survey of hospital management by the PricewaterhouseCoopers Health Research Institute in conjunction with the Healthcare Financial Management Association (HFMA) found that the three most frequently cited capital projects slated for the next five years are all IT acquisitions: 71.7 % for digital radiology systems, 64.1 % for computerised physician order entry systems and 61.3 % for major IT systems [17].

As the sum of the parts is larger than of the sum of each individual piece combined, the benefits of digital image management will only accrue if it can be made available across various platforms (HIS. LIS, PIS, ect) within the same institution, across institutions and ultimately across borders. It is interesting to note that all the disparate digital information systems that make up the Total Hospital Information system (THIS) have been developed independently by the various suppliers for hardware e.g. the Laboratory Information System (LIS) was developed by the people who manufactured and supplied the laboratory instrumentation and it was developed based on the operating software running on all these very different pieces of equipment. As the size, number and complexity of the equipment grew larger, the “fixes” needed became even more mind boggling. Essentially the difficulty of developing common operating standards suggests that the proposed benefits for most institutions is yet to come.

The benefits of information technology in healthcare are widely recognised and have been promoted in industrialised nations against the background of escalating costs of healthcare, increased competitiveness, changing demographics and different disease patterns. However, the relevance of IT in healthcare in developing nations, where the basic healthcare needs have not been met, has not been clearly defined. Very often technology is promoted as the saviour to overcome a myriad of challenges faced by developing nations and technology is seen as an end in itself and not an enabler for a larger purpose. Nations are often seduced into acquiring expensive technology because it is seen as sexy! Or technology is acquired as a marketing tool to convince the public that this is the “happening” community or hospital at the forefront of healthcare!

Would the accrual benefits highlighted for the developed nations be applicable? At what level, and at what cost? What models will be most appropriate for this wide range of development states? What level of technology would be most appropriate? What would be the prerequisites that nations should possess, if information technology in healthcare is to deliver on its potential and promise? Is the promotion of healthcare information technology relevant? Are doctors to be equally blamed for these excesses as a result of creative marketing? We may not have the answers today, but certainly, these are issues that we must come to sooner or later.

## CONCLUSION

The convergence of economics, culture and politics is happening around the globe. Globalisation is causing profound and complex changes in the very nature of our society, bringing new opportunities as well as risks. Therefore, to truly comprehend the interconnected nature of a globalised world, to truly understand the consequences of our policy choices, and to truly grasp the new face of the world, all of us need to understand how globalisation works, what are the policy choices we are facing, and what are the consequences of such choices. While thoughtful, deliberate, and innovative leadership is necessary to help shape globalisation, the process itself is inevitable, even if the final form may be very different.

Globalisation is causing a growing concern for our health, and the intergenerational equity implied by 'sustainable development' forces us to think about the right of future generations to a healthy environment and a healthy life. Even though we are unable to predict the future, we have an opportunity to shape our operating systems and determine the future scope and design of our healthcare systems. The need to balance healthcare costs while providing high-quality care and universal access is nothing less than an exercise in leadership for this 21^st^ century [14].
